# Impact of environmental factors on the emergence, transmission and distribution of *Toxoplasma gondii*

**DOI:** 10.1186/s13071-016-1432-6

**Published:** 2016-03-10

**Authors:** Chao Yan, Li-Jun Liang, Kui-Yang Zheng, Xing-Quan Zhu

**Affiliations:** State Key Laboratory of Veterinary Etiological Biology, Key Laboratory of Veterinary Parasitology of Gansu Province, Lanzhou Veterinary Research Institute, Chinese Academy of Agricultural Sciences, Lanzhou, Gansu Province 730046 PR China; Department of Pathogenic Biology and Immunology, Laboratory of Infection and Immunity, Xuzhou Medical College, Xuzhou, Jiangsu Province 221004 PR China; Department of Clinical Medicine, Xuzhou Medical College, Xuzhou, Jiangsu Province 221004 PR China; Jiangsu Co-innovation Center for the Prevention and Control of Important Animal Infectious Diseases and Zoonoses, Yangzhou University College of Veterinary Medicine, Yangzhou, Jiangsu Province 225009 PR China

**Keywords:** *Toxoplasma gondii*, Ecology, Changing climate, Human activities, Oocysts

## Abstract

*Toxoplasma gondii* is an obligate intracellular protozoan that poses a great threat to human health and economic well-being worldwide. The effects of environmental factors such as changing climate and human activities on the ecology of this protozoan are being discovered. Accumulated evidence shows that changes of these environmental factors can exert influence on the occurrence, transmission and distribution of *T. gondii.* This article reviews studies from different geographical regions with varying climates, social cultures and animal welfare standards. It aims to illustrate how these environmental factors work, highlighting their importance in influencing the ecology of *T. gondii*, as well as providing clues which may contribute to preventing transmission of this important zoonotic pathogen.

## Background

Toxoplasmosis is a globally distributed, water- and food-borne zoonosis caused by the unique protozoan *Toxoplasma gondii*, an organism that infects all warm-blood vertebrates including humans and birds [1]. An estimated one-third of the world’s population is infected by this pathogen [2]. *T. gondii* infects its hosts *via* three main pathways: ingestion of tissue cysts in undercooked meat, ingestion of oocysts in the environment, and congenital transmission from an infected mother to her fetus during pregnancy. Infection with *T. gondii* is typically asymptomatic and causes a life-long latent infection in healthy individuals. A more serious problem is that chronic infection can be reactivated and cause toxoplasmic encephalitis or death in subsequently immunocompromised hosts, such as AIDS patients [3]. Multiple lines of evidence indicate that chronic infections caused by *T. gondii* are likely associated with certain psychiatric disorders in human beings [4, 5]. Furthermore, *T. gondii* infection may result in severe and life-threatening consequences in the developing fetus or newborn, including miscarriage, congenital blindness, hydrocephalus, mental retardation, and even death [6].

Changing environmental conditions, whether caused by rapid urbanization, global warming or economic globalization, have already changed the emergence, transmission and distribution of parasitic diseases including toxoplasmosis [7]. The ecology of *T. gondii,* with its complex life-cycle, is susceptible to environmental change mainly through the survival time and infectivity of oocysts, as well as the behavior and population density of hosts [8]. However, the mechanisms by which these factors affect the spread of *T. gondii* are not fully understood. We therefore reviewed the literature to determine the environmental factors affecting the emergence, transmission and distribution of *T. gondii*.

## The life-cycle of *T. gondii*

*T. gondii* is a protozoan parasite whose complex life-cycle includes sexual replication in the only definitive host, the felid, and asexual propagation in various other warm-blooded animals [1]. When felids ingest foods contaminated by infectious oocysts, tissue cysts or pseudocysts, sporozoites, bradyzoites or tachyzoites are released. The released organisms penetrate the enterocytes of cats and transform intracellularly into multinucleated schizonts after 3 to 7 days post-infection. The subsequent schizogony of sexual development (replication) occurs within the epithelial cells of the small intestine of felids, where schizonts mature and develop into microgametes (male) and macrogametes (female) after several generations [9, 10]. A diploid zygote that will develop into an oocyst forms after a female gamete is fertilized by a male gamete [11]. Unsporulated oocysts are expelled into the environment with the feces in high numbers and become sporulated and infectious; under favourable conditions, an oocyst yields two sporocysts containing four haploid sporozoites [12].

Asexual development begins in the small intestine of hosts when sporulated oocysts in the environment, or the tissue cysts of animal bodies, are ingested by any of a wide range of warm-blood animals, including cats. At first, sporozoites or bradyzoites are released from oocysts or tissues cysts; they penetrate into enterocytes and develop into tachyzoites, which multiply rapidly in any nucleated cells. These protozoans then travel throughout the body *via* blood or lymph, so that tissue cysts form in immunopotent individuals, causing a chronic stage of the asexual cycle. Cysts are mainly distributed in the brain, skeletal muscles and cardiac muscles of hosts [1, 10]. Once ingested by intermediate hosts such as dogs, pigs, rodents or humans, the cysts are digested by proteolytic enzymes in the digestive tract of carnivores. Then bradyzoites are released and differentiate back to the tachyzoite stage as they infect the epithelium of the intestinal lumen and disseminate rapidly within leukocytes, thereby spreading throughout the bodies of their hosts [1, 12]. They then become dormant when the tachyzoites are controlled by interferon-γ and T cell responses of immunopotent individuals [1, 13]. However, this stage of conversion between tachyzoite and bradyzoite shows remarkable flexibility. The success of *T. gondii* as a parasite may reflect that unlike some other important apicomplexan parasites, *T. gondii* can also develop asexually, enabling transmission among warm-blooded intermediate hosts through predation, scavenging, and vertical transmission without involvement of the definitive felid host [14].

## *T. gondii* oocysts in the environment

Unsporaluted oocysts are shed by felids infected by ingestion of any of the three infectious forms: tachyzoites, bradyzoites or oocysts. Unsporaluted oocysts without the capability to infect hosts are less environmentally-resistant, and their lifespan and infectivity are affected by climate conditions, particularly by extremes of temperature and decreased relative humidity [8]. The sporulated oocysts become infective and environmentally-resistant for periods depending on local climatic conditions, up to 12–18 months, and still remain viable stored for at least 54 months at 4 °C in water [15, 16]. It appeared that oocysts are more susceptible to freezing than to higher temperatures, for example, sporulated oocysts can remain infectious in −20 °C whereas they can be killed in 55 °C for 2 min [17, 18]. Low humidity is fatal for oocysts, for example, the oocyst at 19 % relative humidity for 11 days can be killed [19]. In addition, sporulated oocysts with an impermeable outer shell are considered resistant to many chemical disinfectants and physical forces [20, 21].

An infected domestic cat between 4 and 13 days after feeding *T. gondii* tissue cysts can excrete more than 20 million oocysts [22, 23], although the prepatent period of shedding oocysts varies with the stage of *T. gondii* ingested by the cat. For example, the prepatent period for oocyst- or tachyzoite-infected cats (18 days or longer) is longer than that of bradyzoite-infected cats (3–10 days after primary infection) [24]. However, little is known of the amount, the distribution and the density of oocysts in the environment disseminated by wild or domestic felids, which have been responsible for several outbreaks of water-borne toxoplasmosis in humans even at very low doses of oocysts in the environment [25, 26].

Oocyst-containing faeces are habitually buried in humid soil or sand by cats and are small enough to be widely dispersed by water flow. Mechanical hosts that have contact with contaminated soil, water or crops, such as earthworms and amoebae, may also play a role in dissemination. These processes result in worldwide contamination of the environment, making environmental contact an important route of infection for humans and animals [27–29]. Rapid and effective detection of *T. gondii* oocysts from environmental samples remains a challenge, due to lack of sensitive methods, low density in the environment and the inhibitors of assays in these complex environmental matrices. In recent years, PCR-based molecular techniques have been established to detect *T. gondii* oocysts in different matrices, such as water, soil and animal tissues [30–33]. This method has been demonstrated more sensitive and less time consuming than the bioassay, suggesting a wide application in public health [31]. However, a proper procedure for oocyst recovery is critical because of its observable loss during the procedure [34]. The outcomes of these assays may be interpreted with caution because the efficiency of these assays may be affected by the concentration and purification techniques used, the composition of the samples and the properties of the oocysts (e.g., fresh oocyst or not, sporulated or not), as well as the presence of inhibitors of detection assays [25, 35–37].

## Environmental factors influence the occurrence, transmission and distribution of *T. gondii*

Accumulated evidence shows that changes in these environmental factors can strongly influence the occurrence, transmission and distribution of *T. gondii*, especially with today’s rapid urbanization, global warming and economic globalization worldwide. The loss and fragmentation of animal habitat and loss of biodiversity caused by the world’s ever-increasing population and the ensuing changes in land use may disturb the ecological equilibrium, providing opportunities for *T. gondii* to spread into new regions.

### Climatic effects on the ecology of *T. gondii*

Today, climate change is both confirmed and being aggravated by three major components which vary in different places and at different times.

1. Global warming due to the increasing concentration of CO_2_ and other greenhouse gases, such as CH_4_ and N_2_O.
2. Rising temperatures cause the hydrologic cycle to change increasing the total precipitation, creating two main aspects: greater rainfall at higher latitudes and decreased precipitation at lower latitudes.
3. Increased frequency in extreme climatic events [38].

Climate effects influence the occurrence, survival, distribution and transmission of *T. gondii* in three main ways.

1. Whether oocysts can be sporulated or not is partly determined by local climate, particularly temperature and humidity [7].
2. The dynamics of oocysts in the environment are influenced by seasonal precipitation, which affects the river flow that can deliver oocysts from the land to the water, including the ocean, leading to water-borne toxoplasmosis and high levels of coastal contamination with this protozoan parasite [25, 39, 40].
3. Climate may also affect the geographic distribution, population density and migration patterns of rodents, migratory birds and arthropods; animals that act as reservoir hosts or transport hosts and play an important role in the emergence, survival, distribution and transmission of *T. gondii* [41–45].

**Fig. 1:**
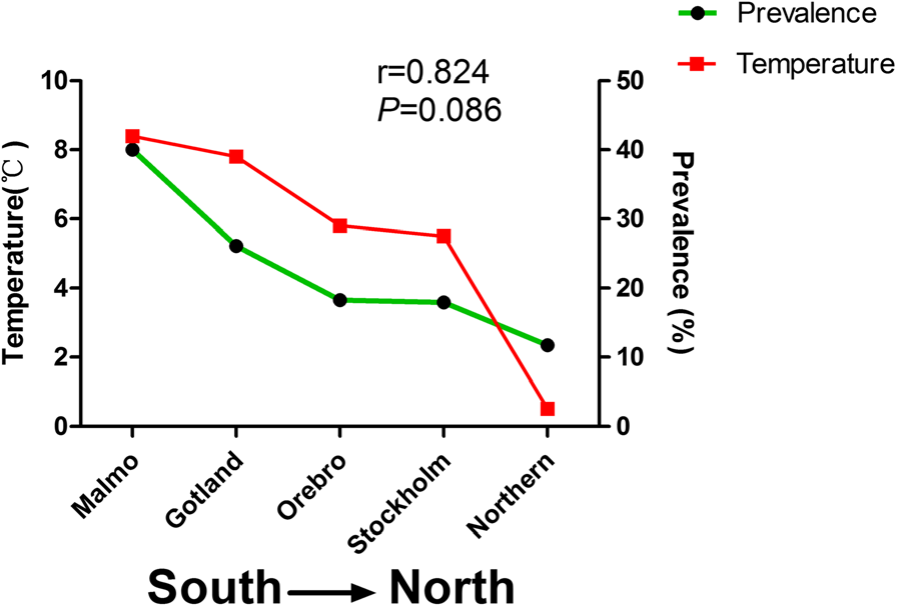
The possible effects of temperature on the prevalence of *Toxoplasma gondii* in pregnant women in Sweden. The data of annual temperature in different regions of Sweden were sourced from the Swedish Meteorological Institute (1961–1990); the data of prevelances of *T. gondii* in pregnant women were obtained from Ref. [48] and Ref. [49]. The correlation was performed using Pearson’s correlation analysis

Temperature may affect the survival and infectivity of *T. gondii* oocysts, as well as the population density of intermediate and mechanical hosts. Even tiny changes in temperature may have a significant effect on the prevalence of the pathogen [46]. For example, in the region of Svalbard, Norway where felids are absent, warmer water temperatures increased the survival time of *T. gondii* and led to influxes of temperate-sensitive marine invertebrate filter feeders such as large marine snails. Moreover, the latter, as carriers for *T. gondii* oocysts, are considered one source of the high seroprevalence of ringed seals and bearded seals, as well as of their predators - polar bears [47]. An interesting study found that the prevalence of toxoplasmosis in pregnant women in Sweden decreased from the south to the north, whereas the average temperature declines along with latitude. To understand further the situation, we analyzed the possible relationship between the prevalence of pregnant women exposed to *T. gondii* and the average annual temperature in the corresponding area using Pearson’s correlation analysis. The result showed a positive correlation between average annual temperature in different areas in Sweden and the incidence of toxoplasmosis in pregnant women, although the *P* value was only 0.086 (Fig. 1) [48, 49]. Another national comparative study in Mexico found that an increase of 0.6 °C in the temperature between 2000 and 2006 was positively correlated with an increased prevalence of toxoplasmosis in humans in 21 states in Mexico (*r* = 0.489, *P* = 0.029) [50]. For terrestrial animals, warmer temperature may increase their abundance, particularly for rodents, which are considered important in the transmission of *T. gondii* as they are prone to predation by pigs, dogs, and particularly by cats [51, 52]. Thus, rising temperatures may provide opportunities for these hosts to increase the dynamic of *T. gondii* and extend its range of distribution [53]. In addition, the increased snowmelt river flow caused by high temperatures during spring conditions could increase the concentration of *T. gondii* oocysts moving from the melting snowpack to the Canadian Arctic estuarine environment, where humans and arctic marine animals are at high risk of infection with *T. gondii* [54, 55].

Rainfall is one of the most important factors influencing the transport of water-borne pathogenic microbial contaminants from the terrestrial environment [56]. The increased river flow caused by increased precipitation in some regions will result in greater delivery of contaminants to the coasts and oceans [7, 57]. One study used logistic regression analysis to find a significant association between *T. gondii* seropositivity in sea otters and sampling areas with a maximal freshwater outflow along the coast, suggesting that freshwater runoff is an important vehicle to transmit *T. gondii* from the land to the marine ecosystem [58, 59]. Furthermore, rain can help create a moist environment that allows for oocysts survival and increase the food availability to support high densities of hosts, including transport hosts [45, 53, 60]. Afonso et al*.* [61] showed that an increased incidence risk of *T. gondii* in cats was found in rainy sites or during rainy years, especially when the mean precipitation per 10-day period was > 25 mm, suggesting that rainfall could influence the exposure of cats to *T. gondii*. Conversely, low rain or drought can result in poor hygiene and reduced food supply, floods in contamination, and the abundance of animals [7]. In addition, alteration to precipitation may affect the development of arthropods that require a humid environment, and flies, cockroaches and earthworms are experimentally-proven transport or paratenic hosts for *T. gondii*, suggesting their potential role in the spread of *T. gondii* to other hosts through ingestion of oocysts [27, 62, 63].

### Human activities

There is little doubt that human activities are changing the environment around us. 1) Climates have been changed by human activities: regional climate can be altered by changes in land use by means of the shifts of surface temperature, radiation and cloudiness; the concentration of atmospheric carbon dioxide (CO_2_) which contributes to global warming has significantly increased due to fossil fuel burning and cement production since the industrial era [38, 64]. 2) Increasing demographics and ensuing changes (including deforestation, urbanization, carbon emissions and other anthropogenic behaviors) have resulted in habitat loss and fragmentation of animal populations, as well as the climate change, which can reduce biological diversity and provide favorable conditions for the occurrence and spread of parasitic zoonosis [7, 65–67]. For example, the emergence of *T. gondii* infections in river otters was likely associated with the higher density of human populations and the increased presence of feral and domestic cats [68]. An increase in the feral cat population responding to the decline of the Tasmanian devil (due to anthropogenic processes) is likely to increase the risk of exposure to *T. gondii* in naive native marsupials in Tasmania, Australia [69]. Gotteland et al*.* developed an Agent Based Model (ABM) taking spatial distribution of landscape structures (farm buildings) into account [70, 71]. They found that spatial distribution of farm buildings in rural environments is associated with the infection of humans and animals: humans and animals in the villages where the farms are located might be at a higher risk of exposure to *T. gondii* oocyst due to the high density of cats [70, 71].

### Urbanization

It is estimated that more than half of the world population was living in cities by 2014 and that the world urban population will increase by another 2.5 billion by 2050, indicating a massive migration from rural to urban, particularly in developing countries [72]. The process of urbanization inevitably alters the land cover and concentrates the population, significantly altering ecological mechanisms that influence the epidemiology of infectious diseases, affecting contact rates in animal and human populations *via* the loss of natural habitat and concentration of domestic and feral animals in urban areas [45, 73–78]. For example, urbanization increases the possibility of host exposure to enteric parasites due to the changes in developed habitat, grassland cover and dietary choice [79]. Lehrer et al. [80] showed that woodchucks in the US Midwest became herbivorous “urban adapters” due to urbanization, and urban woodchucks with a higher prevalence of *T. gondii* shared the same habitat with feral or domestic cats that may discharge high levels of *T. gondii* oocysts into the environment. Lélu et al*.* [81] found that the transmission dynamics of *T. gondii* differed by the type of environment (i.e., rural/urban) and suggested that people living in rural areas tend to have a higher risk of exposure to *T. gondii*, whereas urban dwellers were likely to be infected by *T. gondii* oocysts. Therefore, urbanization may greatly affect the transmission pattern of *T. gondii* and increase the risk of acquiring *T. gondii* infection associated with oocysts. Obviously, further investigation is needed to elucidate the detailed mechanisms of these processes.

### Environmental degradation

Environmental degradation includes deforestation and the degradation of wetlands due to commercial development or agricultural practices and is a global concern [82]. Using a multi-scale, model-based approach, several studies demonstrated that wetlands around human habitation could reduce the load of *T. gondii* oocysts in the coastal environment through the processes of filtering and adhesion [57, 83, 84]. However, the constant degradation of landscapes caused by anthropogenic activity has increased the transmission of zoonotic pathogens to coastal waters, a process likely responsible for widespread infections or death in some species of marine animals that either ingest the contaminated shellfish or are themselves infected by these zoonotic pathogens [81, 85]. A recent study indicated that a significant increase of *T. gondii* in coastal waters threatens the health of marine animals, as well as that of the humans who eat raw or undercooked shellfish, because the degradation of wetlands impairs the ability of plants to eliminate the *T. gondii* oocyst from the surface water in coastal California [84, 86]. However, more investigations are needed to better characterize the relationship between the transmission of *T. gondii* and the ecology ecosystem surrounding us.

## Conclusions

PCR-based molecular approaches used in epidemiological investigations provide powerful tools to monitor and identify the risk factors associated with the spread of *T. gondii* in the environment, especially in determining the sources of infection and the genotypes involved, as well as tracking the transmission route of *T. gondii* directly from environmental matrices [63]. However, advanced monitoring and supervision are needed to enhance the abilities of early-warning systems and prevent outbreaks of toxoplasmosis.

The continued changes in environmental factors have modified the emergence, distribution and transmission of *T. gondii*, although these effects may vary by geographic region according to the local climate, agriculture practice, animal welfare, as well as regional culture. However, those examples mentioned in this review reveal the tremendous gaps in our knowledge of the relationship between the ecology of *T. gondii* and the changes in environmental factors. This knowledge is critical to effectively alleviate and prevent this disease.

Without effective vaccines to prevent human toxoplasmosis, an integrated strategy of prevention should include improved sanitation, changed eating habits, proper handling of cat feces and providing safe drinking water, to reduce or eliminate the transmission of *T. gondii*. Importantly, more efforts should be made to develop collaborations among parasitologists, ecologists, epidemiologists, statisticians, modelers and GIS specialists [87–89]. In the long term, to prevent this opportunistic zoonotic pathogen, effective vaccines must be developed that can block felid oocyst shedding and protect humans from infection, to control toxoplasmosis in humans and animals [90, 91].

## References

[1] Dubey JP (2010). Toxoplasmosis of Animals and Humans.

[2] Tenter AM, Heckeroth AR, Weiss LM (2000). *Toxoplasma gondii*: from animals to humans. Int J Parasitol..

[3] Pereira-Chioccola VL, Vidal JE, Su C (2009). *Toxoplasma gondii* infection and cerebral toxoplasmosis in HIV-infected patients. Future Microbiol..

[4] Fekadu A, Shibre T, Cleare AJ (2010). Toxoplasmosis as a cause for behaviour disorders-overview of evidence and mechanisms. Folia. Parasitol. Praha..

[5] Parlog A, Schlüter D, Dunay IR (2015). *Toxoplasma gondii*-induced neuronal alterations. Parasite Immunol..

[6] Galvan-Ramírez Mde L, Troyo-Sanroman R, Roman S, Bernal-Redondo R, Vázquez Castellanos JL. Prevalence of *toxoplasma* infection in Mexican newborns and children: a systematic review from 1954 to 2009. ISRN Pediatr. 2012;2012:501216. 10.5402/2012/501216 PMC3463180 23050161

[7] Patz JA, Graczyk TK, Geller N, Vittor AY (2000). Effects of environmental change on emerging parasitic diseases. Int J Parasitol..

[8] Meerburg BG, Kijlstra A (2009). Changing climate-changing pathogens: *Toxoplasma gondii* in North-Western Europe. Parasitol Res..

[9] Speer CA, Dubey JP (2005). Ultrastructural differentiation of *Toxoplasma gondii* schizonts (types B to E) and gamonts in the intestines of cats fed bradyzoites. Int J Parasitol..

[10] Dubey JP, Frenkel JK (1972). Cyst-induced toxoplasmosis in cats. J Protozool..

[11] Ferguson DJ, Hutchison WM, Siim JC (1975). The ultrastructural development of the macrogamete and formation of the oocyst wall of *Toxoplasma gondii*. Acta Pathol Microbiol Scand B..

[12] Dubey JP, Miller NL, Frenkel JK (1970). The *Toxoplasma gondii* oocyst from cat feces. J Exp Med..

[13] Gross U, Bohne W, Soête M, Dubremetz JF (1996). Developmental differentiation between tachyzoites and bradyzoites of *Toxoplasma gondii*. Parasitol Today..

[14] Su C, Evans D, Cole RH, Kissinger JC, Ajioka JW, Sibley LD (2003). Recent expansion of *Toxoplasma* through enhanced oral transmission. Science..

[15] Yilmaz SM, Hopkins SH (1972). Effects of different conditions on duration of infectivity of *Toxoplasma gondii* oocysts. J Parasitol..

[16] Lindsay DS, Dubey JP (2009). Long-term survival of *Toxoplasma gondii* sporulated oocysts in seawater. J Parasitol..

[17] Frenkel JK, Dubey JP (1973). Effects of freezing on the viability of toxoplasma oocysts. J Parasitol..

[18] Dubey JP (1998). *Toxoplasma gondii* oocyst survival under defined temperatures. J Parasitol..

[19] Frenkel JK, Dubey JP (1972). Toxoplasmosis and its prevention in cats and man. J Infect Dis.

[20] Wainwright KE (2007). Chemical inactivation of *Toxoplasma gondii* oocysts in water. J Parasitol..

[21] Dumètre A (2008). Effects of ozone and ultraviolet radiation treatments on the infectivity of *Toxoplasma gondii* oocysts. Vet Parasitol..

[22] Dubey JP (1995). Duration of immunity to shedding of *Toxoplasma gondii* oocysts by cats. J Parasitol..

[23] Dubey JP (2001). Oocyst shedding by cats fed isolated bradyzoites and comparison of infectivity of bradyzoites of the VEG strain *Toxoplasma gondii* to cats and mice. J Parasitol..

[24] Dubey JP (2005). Unexpected oocyst shedding by cats fed *Toxoplasma gondii* tachyzoites: in vivo stage conversion and strain variation. Vet Parasitol..

[25] Karanis P, Aldeyarbi HM, Mirhashemi ME, Khalil KM (2013). The impact of the waterborne transmission of *Toxoplasma gondii* and analysis efforts for water detection: an overview and update. Environ Sci Pollut Res Int..

[26] Dubey JP, Lunney JK, Shen SK, Kwok OC, Ashford DA, Thulliez P (1996). Infectivity of low numbers of *Toxoplasma gondii* oocysts to pigs. J Parasitol..

[27] Bettiol SS, Obendorf DL, Nowarkowski M, Milstein T, Goldsmid JM (2000). Earthworms as paratenic hosts of toxoplasmosis in eastern barred bandicoots in Tasmania. J Wildl Dis..

[28] Frenkel JK, Lindsay DS, Parker BB, Dobesh M (2003). Dogs as possible mechanical carriers of *Toxoplasma*, and their fur as a source of infection of young children. Int J Infect Dis..

[29] Winiecka-Krusnell J (2009). *Toxoplasma gondii*: uptake and survival of oocysts in free-living amoebae. Exp Parasitol..

[30] Palos Ladeiro M, Bigot-Clivot A, Aubert D, Villena I, Geffard A (2015). Assessment of *Toxoplasma gondii* levels in zebra mussel (*Dreissena polymorpha*) by real-time PCR: an organotropism study. Environ Sci Pollut Res Int..

[31] Aubert D, Villena I (2009). Detection of *Toxoplasma gondii* oocysts in water: proposition of a strategy and evaluation in Champagne-Ardenne Region, France. Mem Inst Oswaldo Cruz..

[32] Shapiro K, VanWormer E, Aguilar B, Conrad PA (2015). Surveillance for *Toxoplasma gondii* in California mussels (*Mytilus californianus*) reveals transmission of atypical genotypes from land to sea. Environ Microbiol..

[33] Lélu M, Gilot-Fromont E, Aubert D, Richaume A, Afonso E, Dupuis E (2011). Development of a sensitive method for *Toxoplasma gondii* oocyst extraction in soil. Vet Parasitol..

[34] Nieminski EC (1995). Comparison of two methods for detection of *Giardia* cysts and *Cryptosporidium* oocysts in water. Appl Environ Microbiol..

[35] Dumètre A (2004). Purification of *Toxoplasma gondii* oocysts by cesium chloride gradient. J Microbiol Methods..

[36] Kourenti C, Karanis P (2006). Evaluation and applicability of a purification method coupled with nested PCR for the detection of *Toxoplasma* oocysts in water. Lett Appl Microbiol..

[37] Zilberman A (2009). A Two-Phase Separation Method for Recovery of *Cryptosporidium* Oocysts from Soil Samples. Water Air Soil Pollut..

[38] Intergovernmental Panel on Climate Change. https://www.ipcc.ch/pdf/assessment-report/ar5/syr/AR5_SYR_FINAL_SPM.pdf. (2014). Accessed 17 August 2015.

[39] Mazzillo FF, Shapiro K, Silver MW (2013). A New Pathogen Transmission Mechanism in the Ocean: The Case of Sea Otter Exposure to the Land-Parasite *Toxoplasma gondii*. PLoS One..

[40] Ribeiro LA, Santos LK, Brito PA, Maciel BM, Da Silva AV, Albuquerque GR (2015). Detection of *Toxoplasma gondii* DNA in Brazilian oysters *(Crassostrea rhizophorae*). Genet Mol Res..

[41] Root TL (2003). Fingerprints of global warming on wild animals and plants. Nature..

[42] Prestrud KW, Asbakk K, Fuglei E, Mørk T, Stien A, Ropstad E (2007). Serosurvey for *Toxoplasma gondii* in arctic foxes and possible sources of infection in the high Arctic of Svalbard. Vet Parasitol..

[43] Dhimal M, Ahrens B, Kuch U (2014). Species composition, seasonal occurrence, habitat preference and altitudinal distribution of malaria and other disease vectors in eastern Nepal. Parasit Vectors..

[44] Elmore SA, Jenkins EJ, Huyvaert KP, Polley L, Root JJ, Moore CG (2012). *Toxoplasma gondii* in circumpolar people and wildlife. Vector Borne Zoonotic Dis..

[45] Afonso E, Germain E, Poulle ML, Ruette S, Devillard S, Say L (2013). Environmental determinants of spatial and temporal variations in the transmission of *Toxoplasma gondii* in its definitive hosts. Int J Parasitol Parasites Wildl..

[46] Laaksonen S (2010). Climate change promotes the emergence of serious disease outbreaks of filarioid nematodes. Ecohealth..

[47] Jensen SK (2010). The prevalence of *Toxoplasma gondii* in polar bears and their marine mammal prey: evidence for a marine transmission pathway?. Polar Biology..

[48] Ljungström I, Gille E, Nokes J, Linder E, Forsgren M (1995). seroepidemiology of *Toxoplasma gondii* among pregnant women in different parts of sweden. Eur J Epidemiol.

[49] Ahlfors K, Börjeson M, Huldt G, Forsberg E (1989). Incidence of toxoplasmosis in pregnant women in the city of Malmö Sweden. Scand J Infect Dis..

[50] Caballero-Ortega H, Uribe-Salas FJ, Conde-Glez CJ, Cedillo-Pelaez C, Vargas-Villavicencio JA, Luna-Pastén H (2012). Seroprevalence and national distribution of human toxoplasmosis in Mexico: analysis of the 2000 and 2006 National Health Surveys. Trans R Soc Trop Med Hyg..

[51] Kijlstra A, Meerburg B, Cornelissen J, De Craeye S, Vereijken P, Jongert E (2008). The role of rodents and shrews in the transmission of *Toxoplasma gondii* to pigs. Vet Parasitol..

[52] Jiang W, Sullivan AM, Su C, Zhao X (2012). An agent-based model for the transmission dynamics of *Toxoplasma gondii*. J Theor Biol..

[53] Gubler DJ, Reiter P, Ebi KL, Yap W, Nasci R, Patz JA (2001). Climate variability and change in the United States: potential impacts on vector- and rodent-borne diseases. Environ Health Perspect..

[54] Simon A, Poulin MB, Rousseau AN, Ogden NH (2013). Fate and Transport of *Toxoplasma gondii* oocysts in seasonally snow covered watersheds: A conceptual framework from a melting snowpack to the Canadian Arctic Coasts. Int J Environ Res Public Health..

[55] Simon A, Rousseau AN, Savary S, Bigras-Poulin M, Ogden NH (2013). Hydrological modelling of *Toxoplasma gondii* oocysts transport to investigate contaminated snowmelt runoff as a potential source of infection for marine mammals in the Canadian Arctic. J Environ Manage..

[56] Gotteland C, Gilot-Fromont E, Aubert D, Poulle ML, Dupuis E, Dardé ML (2014). Spatial distribution of *Toxoplasma gondii* oocysts in soil in a rural area: Influence of cats and land use. Vet Parasitol..

[57] Shapiro K, Miller M, Mazet J (2012). Temporal association between land-based runoff events and California sea otter (*Enhydra lutris nereis*) protozoal mortalities. J Wildl Dis..

[58] Miller MA, Gardner IA, Kreuder C, Paradies DM, Worcester KR, Jessup DA (2002). Coastal freshwater runoff is a risk factor for *Toxoplasma gondii* infection of southern sea otters (*Enhydra lutris nereis*). Int J Parasitol..

[59] Jasper JT, Nguyen MT, Jones ZL, Ismail NS, Sedlak DL, Sharp JO (2013). Unit process wetlands for removal of trace organic contaminants and pathogens from municipal wastewater effluents. Environ Eng Sci..

[60] Stenseth CN, VIljugrein H, Jędrzejewski W, Mysterud A, Pucek Z (2002). Population dynamics of *Clethrionomys glareolus* and *Apodemus flavicollis*: seasonal components of density dependence and density independence. Acta Theriol..

[61] Afonso E, Thulliez P, Gilot-Fromont E (2010). Local meteorological conditions, dynamics of seroconversion to *Toxoplasma gondii* in cats (*Felis catus*) and oocyst burden in a rural environment. Epidemiol Infect.

[62] Frenkel JK, Hassanein KM, Hassanein RS, Brown E, Thulliez P, Quintero-Nunez R (1995). Transmission of *Toxoplasma gondii* in Panama City, Panama: a five-year prospective cohort study of children, cats, rodents, birds, and soil. Am J Trop Med Hyg..

[63] Polley L, Thompson RC (2009). Parasite zoonoses and climate change: molecular tools for tracking shifting boundaries. Trends Parasitol..

[64] de la Rocque S (2008). Climate change: impact on the epidemiology and control of animal diseases. Introduction Rev Sci Tech..

[65] Macpherson CN (2005). Human behavior and the epidemiology of parasitic zoonoses. Int J Parasitol..

[66] Patz JA (2008). Disease emergence from global climate and land use change. Med Clin North Am..

[67] Mbora DN, McPeek MA (2009). Host density and human activities mediate increased parasite prevalence and richness in primates threatened by habitat loss and fragmentation. J Anim Ecol..

[68] Gaydos JK, Conrad PA, Gilardi KV, Blundell GM, Ben-David M (2007). Does human proximity affect antibody prevalence in marine-foraging river otters (*Lontra canadensis*)?. J Wildl Dis..

[69] Hollings T, Jones M, Mooney N, McCallum H (2013). Wildlife disease ecology in changing landscapes: Mesopredator release and toxoplasmosis. Int J Parasitol Parasites Wildl..

[70] Gotteland C, McFerrin BM, Zhao X, Gilot-Fromont E, Lélu M (2014). Agricultural landscape and spatial distribution of *Toxoplasma gondii* in rural environment: an agent-based model. Int J Health Geogr..

[71] Gotteland C, Chaval Y, Villena I, Galan M, Geers R, Aubert D (2014). Species or local environment, what determines the infection of rodents by *Toxoplasma gondii*?. Parasitology..

[72] United Nations. http://www.un.org/en/development/desa/publications/2014-revision-world-urbanization-prospects.html (2014). Accessed 10 July 2014.

[73] Afonso E, Lemoine M, Poulle ML, Ravat MC, Romand S, Thulliez P (2008). Spatial distribution of soil contamination by *Toxoplasma gondii* in relation to cat defecation behavior in an urban area. Int J Parasitol..

[74] Sepúlveda MA, Muñoz-Zanzi C, Rosenfeld C, Jara R, Pelican KM, Hill D (2011). *Toxoplasma gondii* in feral American minks at the Maullín river, Chile. Vet Parasitol..

[75] Yan C, Fu LL, Yue CL, Tang RX, Liu YS, Lv L (2012). Stray dogs as indicators of *Toxoplasma gondii* distributed in the environment: the first report across an urban–rural gradient in China. Parasit Vectors.

[76] Ferreira JP, Leitão I, Santos-Reis M, Revilla E (2011). Human-Related Factors Regulate the Spatial Ecology of Domestic Cats in Sensitive Areas for Conservation. PLoS One..

[77] Neiderud CJ (2015). How urbanization affects the epidemiology of emerging infectious diseases. Infect Ecol Epidemiol..

[78] Nijsse R, Mughini-Gras L, Wagenaar JA, Franssen F, Ploeger HW (2015). Environmental contamination with *Toxocara* eggs: a quantitative approach to estimate the relative contributions of dogs, cats and foxes, and to assess the efficacy of advised interventions in dogs. Parasit Vectors..

[79] Watts AG, Lukasik VM, Fortin MJ, Alexander SM (2015). Urbanization, grassland, and diet influence coyote (*Canis latrans*) parasitism structure. Ecohealth..

[80] Lehrer EW, Fredebaugh SL, Schooley RL, Mateus-Pinilla NE (2010). Prevalence of antibodies to *Toxoplasma gondii* in woodchucks across an urban–rural gradient. J Wildl Dis..

[81] Lélu M, Langlais M, Poulle ML, Gilot-Fromont E (2010). Transmission dynamics of *Toxoplasma gondii* along an urban–rural gradient. Theor Popul Biol..

[82] Scavia D, Field CJ, Boesch FD, Buddemeier WR, Burkett V, Cayan RD (2002). Climate change impacts on US coastal and marine ecosystems. Estuaries..

[83] Daniels ME, Hogan J, Smith WA, Oates SC, Miller MA, Hardin D (2014). Estimating environmental conditions affecting protozoal pathogen removal in surface water wetland systems using a multi-scale, model-based approach. Sci Total Environ..

[84] Hogan JN, Daniels ME, Watson FG, Oates SC, Miller MA, Conrad PA (2013). Hydrologic and vegetative removal of *Cryptosporidium parvum*, *Giardia lamblia*, and *Toxoplasma gondii* surrogate microspheres in coastal wetlands. Appl Environ Microbiol..

[85] Fayer R (2004). Zoonotic protozoa: from land to sea. Trends Parasitol..

[86] Shapiro K, Conrad PA, Mazet JA, Wallender WW, Miller WA, Largier JL (2010). Effect of estuarine wetland degradation on transport of *Toxoplasma gondii* surrogates from land to sea. Appl Environ Microbiol..

[87] Zhou P, Chen Z, Li HL, Zheng H, He S, Lin RQ (2011). *Toxoplasma gondii* infection in humans in China. Parasit Vectors..

[88] Rohr JR, Dobson AP, Johnson PT, Kilpatrick AM, Paull SH (2011). Frontiers in climate change-disease research. Trends Ecol Evol..

[89] VanWormer E, Fritz H, Shapiro K, Mazet JA, Conrad PA (2013). Molecules to modeling: *Toxoplasma gondii* oocysts at the human-animal-environment interface. Comp Immunol Microbiol Infect Dis..

[90] Zhang NZ, Chen J, Wang M, Petersen E, Zhu XQ (2013). Vaccines against *Toxoplasma gondii*: new developments and perspectives. Expert Review Vaccines..

[91] Zhang NZ, Wang M, Xu Y, Petersen E, Zhu XQ (2015). Recent advances in developing vaccines against *Toxoplasma gondii*: an update. Expert Rev Vaccines..

